# Prenatal Pollutant Exposures and Hypothalamic Development: Early Life Disruption of Metabolic Programming

**DOI:** 10.3389/fendo.2022.938094

**Published:** 2022-07-11

**Authors:** Lisa Koshko, Sydney Scofield, Gil Mor, Marianna Sadagurski

**Affiliations:** ^1^Integrative Biosciences Center, Department of Biological Sciences, Wayne State University, Detroit, MI, United States; ^2^C.S. Mott Center for Human Growth and Development, Department of Obstetrics and Gynecology School of Medicine, Wayne State University, Detroit, MI, United States

**Keywords:** prenatal environmental exposures, air pollution, hypothalamic development, neuroinflammation, metabolic programming, metabolic syndrome, diabetes

## Abstract

Environmental contaminants in ambient air pollution pose a serious risk to long-term metabolic health. Strong evidence shows that prenatal exposure to pollutants can significantly increase the risk of Type II Diabetes (T2DM) in children and all ethnicities, even without the prevalence of obesity. The central nervous system (CNS) is critical in regulating whole-body metabolism. Within the CNS, the hypothalamus lies at the intersection of the neuroendocrine and autonomic systems and is primarily responsible for the regulation of energy homeostasis and satiety signals. The hypothalamus is particularly sensitive to insults during early neurodevelopmental periods and may be susceptible to alterations in the formation of neural metabolic circuitry. Although the precise molecular mechanism is not yet defined, alterations in hypothalamic developmental circuits may represent a leading cause of impaired metabolic programming. In this review, we present the current knowledge on the links between prenatal pollutant exposure and the hypothalamic programming of metabolism.

## Introduction

**Figure 1:**
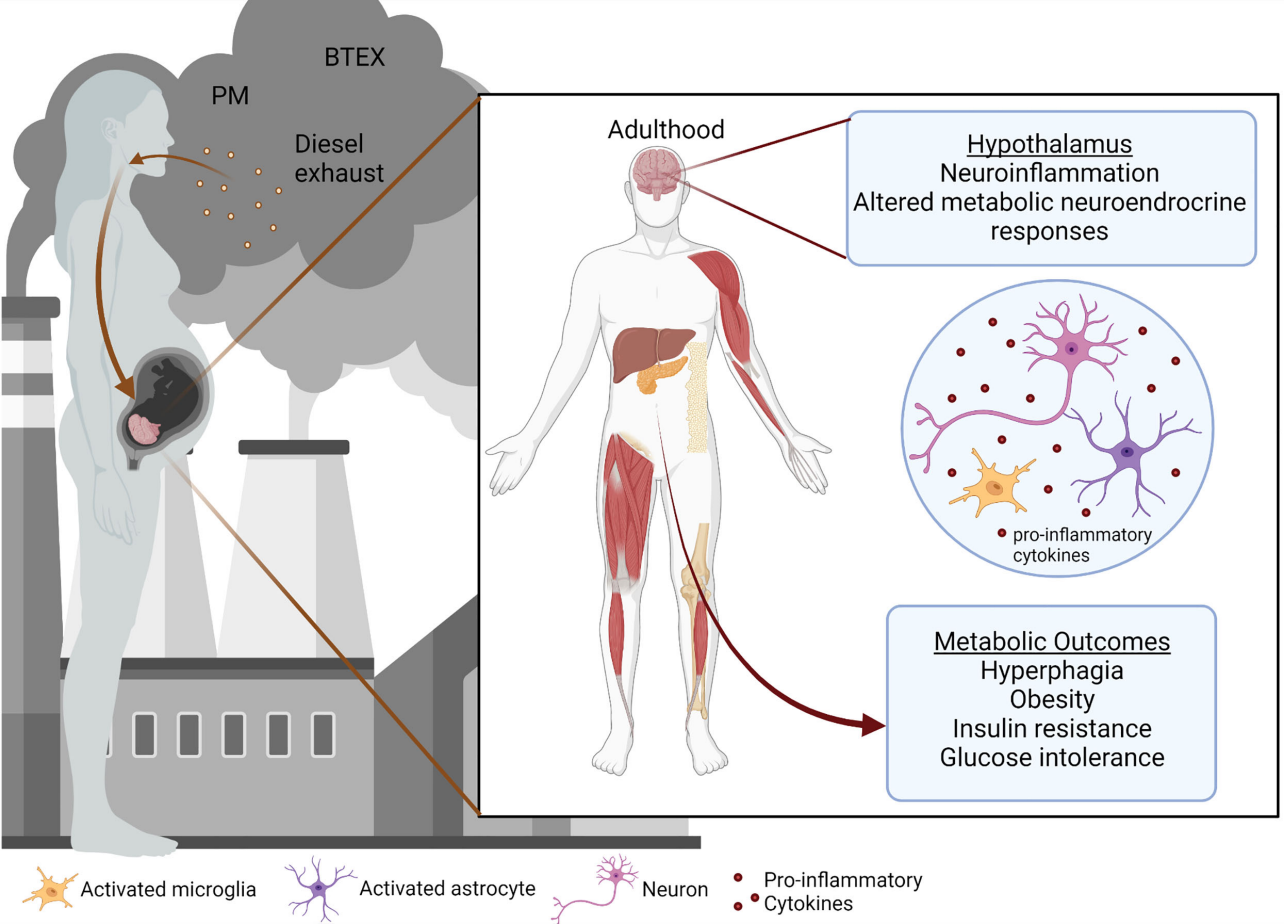
Prenatal air pollution exposure induces hypothalamic and metabolic dysfunction.

Air pollution is one of the leading environmental concerns and poses a significant risk to the health of people around the world, despite advancements in medicine and technology. According to the World Health Organization, around 7 million deaths were prematurely caused by air pollution per year, including both ambient outdoor pollution and household pollution (1, 2). Of those deaths in 2016, the majority (4.2 million) were caused by outdoor air pollution including particulate matter (PM) (3, 4), ozone, nitrogen and sulfur dioxide, and carbon monoxide (5). Exposures to air pollution during early life and adulthood have been shown to propagate adverse health effects (6–13). Still, less is known about the impact of early-life exposures during gestation and the neonatal period on metabolic syndrome (14). A growing body of literature suggests that environmental contaminants can predispose to metabolic syndrome and disease, which have steadily increased in recent decades and are projected to continue rising (8, 15–20). While an exact mechanism linking pollutant exposures with metabolic programming remains unclear, a combination of factors likely determines the predisposition to impaired metabolism. Here we discuss a few of the possible routes by which air pollution could be contributing to metabolic disruption in offspring (**Figure 1**).

## The Developmental Programming of the Hypothalamus

In the CNS, the hypothalamus is the main region critical for the regulation of whole-body metabolism (21, 22). The hypothalamus is comprised of nuclei containing distinct neuronal populations that produce neuropeptides critical for the regulation of body core temperature, metabolic rate, satiety signals, sexual dimorphism and reproduction, circadian rhythm, energy homeostasis, and glucose metabolism (22–24). Recent studies in vertebrate genetic models have demonstrated that the development of hypothalamic neurocircuitry can be influenced by various nutritional and environmental cues in early life (25, 26). In humans, connectivity of a subset of these pathways occurs during gestation, while in rodents, refinement of connections occurs in early postnatal life (25). The rodent hypothalamus develops during a relatively long period, beginning early in gestation and continuing during the postnatal period (27). The developing hypothalamus is therefore exposed to two distinct environments: one *in utero* (around mid-gestation to birth) and the other extra utero (27–29). These developmental windows represent important intervals of vulnerability during which alterations in the maternal environment may lead to abnormal hypothalamic development and subsequent metabolic alterations.

The arcuate nucleus of the hypothalamus (ARC) contains pro-opiomelanocortin (POMC) neurons that produce the anorexigenic peptide melanocyte-stimulating hormone (MSH) and neurons that co-express the orexigenic peptides neuropeptide Y (NPY) and agouti-related protein (AgRP), which regulate food intake and energy expenditure (30–32). The POMC and NPY/AgRP neurons project to the paraventricular hypothalamic nucleus (PVN) and lateral hypothalamus (LH), as well as regions outside the hypothalamus, to regulate energy homeostasis and nutrient intake (22). Developmental abnormalities of these hypothalamic neurocircuits are associated with alterations in body weight, metabolic imbalance, chronic stress, and obesity (33). Importantly, the interaction of hypothalamic neurons with neighboring glial cells (especially astrocytes and microglia) is critical for sensing hormonal changes and various metabolites. Impairments in these interactions can have an impact on hypothalamic physiology and dysfunction in the context of systemic metabolism and metabolic disease.

## Glial Role in Hypothalamic Development

Microglia, the resident parenchymal myeloid cells of the CNS, have been shown to play a vital role in hypothalamic development (34). Microglia are remarkably sensitive to external environmental stressors such as ozone, diesel exhaust, air pollution, and environmental contaminants (35–38) (39), causing them to interact with neighboring neurons to control their local environment through the modulation of inflammatory pathways (40–42). During both prenatal and postnatal development, microglia play a critical role in cross-talk between the nervous and immune systems and in many developmental processes (43). Activation of the immune system during pregnancy or early life has been shown to exert long-term effects on the wiring of neural circuits and may contribute to the etiology of neurodevelopmental and metabolic disorders (44–46). In humans, microglia colonize the developing brain between weeks 4 and 24 of gestation (47) while in rodents, it begins around embryonic day 8 (E8) (48). By birth, microglia normally transition from an amoeboid to a ramified “surveillant” state and remain this way until subjected to an immune challenge (49). Maternal exposure to persistent stressors during pregnancy can lead to maternal immune activation (MIA), forcing fetal microglia to remain activated, also known as microglial priming (50, 51). Upon subsequent immune challenges later in life, these cells can inappropriately react with excessive cytokine release as a result of immune memory (51). The early embryonic development of immune-sensing microglia potentially plays a role in the sensitivity of the developing CNS (52). The distribution and function of embryonic microglia in the developing brain was covered in detail elsewhere (53). While the role of microglia in the developmental stages of hypothalamic neurocircuits is still emerging, embryonic microglia can influence gliogenesis within the developing hypothalamus (54). Specific depletion of microglia in mice during embryonic development caused a decrease in hypothalamic POMC neurons postnatally and accelerated weight gain in early postnatal life (34), emphasizing the necessity of microglia for the development of the hypothalamic satiety signals. Hypothalamic embryonic microglia are very sensitive to insults and can coordinate innate immune response following an insult *via* microglial TAM receptors (55), providing additional insights into the role of microglia in hypothalamic developmental programing.

Astrocytes, the most abundant glial cell type in the brain, are largely produced during gliogenesis (53). Astrocyte development begins around E18 and lasts until roughly P7 in mice, although adult astrocytes retain the ability to divide and differentiate (53, 56). Microglia have been proposed to influence the transition from neurogenesis to astrogenesis (57). Like microglia, astrocytes significantly regulate synaptogenesis, mostly postnatally in mice, by secreting factors such as brain-derived neurotrophic factor (BDNF) and glypican 4 and 6 (Gpc4 & Gpc6) and through the generation of lipids (58–60). Under normal physiologic conditions, astrocytes support the nutritional needs of the neurons by producing and shuttling metabolites such as lactate and ketone bodies (61, 62). More recently, astrocytes have been proposed to help maintain the integrity of the blood-brain barrier (63, 64) and synaptic transmission between neurons through the protection of gap junctions (64). Hypothalamic astrocytes sense glucose and fatty acids and express receptors for several peripheral hormones such as leptin and insulin (65). During development, hypothalamic astrocytes express unique clusters of genes critical for growth and development (66). Microglia and astrocytes are in constant crosstalk, thereby influencing the activity of one another. Early-life microglial activation as a result of pollution exposure may thereby alter astrocyte function later in life (67), inducing the activation of astrocytes and microglia and subsequent neuroinflammation (58). Considering the critical roles microglia and astrocytes play during hypothalamic development, understanding the interaction between these cells and their responsiveness to the early-life insults, can provide insights into the pathogenesis of metabolic disease.

## Hypothalamic Response to Pollutants: Neuroinflammation and Altered Development

A growing body of evidence now implicates that exposure to air pollutants and toxins leads to hypothalamic neuroinflammation and subsequent metabolic dysregulation (55, 68–71). For example, when pregnant mice were exposed to diesel exhaust (DE) inhalation from E9-17, the fetal brains of the offspring showed altered cytokine and chemokine levels at E18, including increased pro-inflammatory IL-6 and decreased anti-inflammatory IL-10 (71). In adulthood, DE-exposed offspring fed a high-fat diet (HFD) had increased microglial activation in several brain regions, including the hypothalamus, indicative of long-term microglial priming from the prenatal exposure (71). Additionally, DE-exposed offspring demonstrated increased weight gain, energy intake, and insulin levels, either before or after HFD feeding, with males exhibiting a more severe phenotype (71). Thus, prenatal DE exposure triggers neuroinflammatory responses during gestation that lead to microglial priming, predisposing offspring to adult diet-induced metabolic imbalance and neuroinflammation (71, 72). Similarly, male offspring of pregnant dams treated with intermittent doses of diesel exhaust particles (DEP) from E2-17 demonstrated increased expression of IL-1β in serum and brain tissue following an immune challenge with LPS (69). However, only male offspring of DEP-exposed dams exhibited exaggerated weight gain, insulin resistance, and anxiety-like behavior when challenged with HFD compared with male control offspring (69). In support, we have previously demonstrated that maternal exposure to inhaled benzene throughout pregnancy was associated with hyperglycemia, insulin resistance, reduced energy expenditure, and increased hepatic inflammation in the adult male offspring (73). Similarly, exposure to benzene in adulthood was also associated with a metabolic imbalance in male but not female mice (74).

Harmful environmental conditions can pose a serious threat to the development of hypothalamic neurocircuits (75–80). Exposing rats to various endocrine-disrupting polychlorinated biphenyls (PCBs) during gestation (77) and a subsequent postnatal immune challenge (78), alters hypothalamic neuropeptide gene expression and cytokine levels in the serum in a sexually dimorphic manner (78). During gestation, hypothalamic microglia also show strong sensitivity to exposure to the endocrine-disrupting chemical (EDC) bisphenol A (BPA). In mouse studies, offspring prenatally exposed to BPA had early hypothalamic neurogenesis (81), altered embryonic microglia (82), reduced anorexigenic hypothalamic projections, central leptin resistance, and a delayed postnatal leptin surge (79). Similarly, BPA exposure in pregnant dams induced a significant increase in microglia numbers and the expression of inflammatory genes in the fetal hypothalamus (83). Thus, gestational BPA exposure in mice negatively impacts the development of embryonic hypothalamic microglia, associated with increased microglia numbers, expanded microglial process ramification, and increased numbers of microglial phagocytic cups (82). Studies in rats have also demonstrated the influence of exposure to toxins on postnatal hypothalamic development through lactation (84–86). A recent review presented a series of experiments where rats were exposed to a nicotine level equivalent to heavy smokers during lactation (84). Nicotine-exposed male offspring exhibited increased body weight, adiposity, insulin resistance, and central leptin resistance in adulthood (86, 87). However, the time frame and the route of exposure may differentially impact the metabolic outcomes in young animals and animals exposed to nicotine in adulthood (88). At PN180, nicotine-exposed male offspring had increased expression of α-MSH, corticotrophin-releasing hormone (CRH), and NPY along with decreased cocaine- and amphetamine-regulated transcript (CART) in the PVN (86). Additionally, nicotine-exposed offspring had increased hypothalamic microgliosis and astrogliosis (84, 89). When offspring were exposed to cigarette smoke during lactation, this resulted in impaired development of hypothalamic circuits leading to hyperphagia, obesity, and neuroinflammation in the adult offspring (85).

## Mechanisms Linking Hypothalamic Metabolic Programming and Pollution

How can air pollution and specific particles exert deleterious effects on the hypothalamus during development? It is becoming increasingly accepted that pollution triggers an inflammatory response in peripheral tissues that is associated with an elevation in cytokine secretion. In turn, circulating cytokines produced in systemic inflammation can enter the brain, causing neuroinflammation and neurotoxicity (90, 91).

Maternal inflammation and maternal immune activation (MIA) are known to be harmful to a developing fetus (44, 92–96). A recent study indicates that exposure of pregnant African American women to air pollution was associated with inflammation in the mothers by mid-pregnancy (97). This study focused on ambient exposure to BTEX (benzene, toluene, ethylbenzene, and xylene) and measured maternal inflammatory markers during the second trimester. A positive association was found between the levels of BTEX exposure and inflammatory cytokines IL-1β and TNF-α (97). Maternal exposure to benzene during pregnancy was found to be associated with low birth weight and head circumference (98–100). As shown in rodent models, gestational immune activation can disrupt hypothalamic neurocircuits of maternal care behavior (101), alter the hypothalamic epigenome in the offspring (102), and decrease hypothalamic dopamine neurotransmission (103). Additionally, evidence from rodents has shown that maternal inflammation can result in altered offspring metabolism, such as increased food intake, body weight, and impaired insulin sensitivity (104). Thus, it is likely that maternal exposure to pollution *via* alterations in hypothalamic developmental circuits may contribute to metabolic disease in the offspring.

A key factor that must be considered when determining how the maternal environment influences the offspring is the placenta. The placenta is a vital organ that acts to provide a supportive and protective environment for the developing fetus and as a point of interaction between the mother and fetus (105). However, while some molecules are not able to cross the placenta and act directly on the fetus, they can potentially exert indirect influence *via* inflammation or hypoxia (106). Modulation of placental function by maternal inflammation could, in turn, alter the environment of the fetus and possibly impact its development (107). A pilot study looking at the effects of household air pollution (HAP) on pregnant Nigerian women found that exposure to air pollution was associated with increased markers of chronic hypoxia in the placenta, which was implicated as a mechanism for adverse pregnancy outcomes associated with HAP (108). Conversely, molecules with the ability to cross the placenta can directly interact with the fetus and lead to adverse health effects on brain development (106). Various toxins and chemicals can cross the placenta, as indicated by measurable levels in umbilical cord serum, including multiple organohalogen compounds (OHCs) such as polybrominated diphenyl ethers (PBDEs), phencyclidine (PCP), and polychlorinated biphenyls (PCBs) (109, 110). Other chemicals with the known ability for transfer are BPAs, nicotine from tobacco smoke, phthalate monoesters, and the polycyclic aromatic hydrocarbon (PAH) benzo(a)pyrene (81, 82, 111). BPAs have been found in human placental tissue, umbilical cord blood, and fetal plasma (112–114). Volatile organic compounds (VOCs) such as benzene, ethylbenzene, xylene, carbon tetrachloride, and chloroform can also cross the placenta during pregnancy and have an impact on the developing fetus (115). Once the pollutants and particles reach the developing brain, there is considerable debate as to what are the precise mechanisms of toxicity. One potential mechanism by which gestational exposure to pollutants may cause impaired health outcomes is *via* neuroinflammation mediated by the activation of the brain’s innate immune system in response to an inflammatory challenge, which leads to adverse neural adaptations and neurotoxicity (40, 41, 116). Developmental abnormalities in the hypothalamus and neuroendocrine system induced by air pollution (117) and the stimulated innate immunity in the brain can provide a potential mechanistic link for peripheral chronic disease susceptibility.

Given the chronic nature of human exposure to environmental toxins over an entire lifetime, including the critical periods of hypothalamic development, this could alter later life metabolism, contributing to metabolic disease (29). Although there is a lack of information on the hypothalamic consequences of pollutant exposure in humans, epidemiological studies indicate that air pollution increases the risk of metabolic disease, which may be worsened by poor lifestyle choices such as lack of exercise, alcohol consumption, and obesity (118). Observational studies in humans have linked exposures to various pollutants including PM 2.5 and ozone with higher rates of T2DM in populations across the globe (119–123). Healthy mothers living near busy streets at preconception had increased fasting blood glucose levels, suggesting that air pollution exposure contributes to metabolic imbalance (124). Disturbances in hypothalamic development could result in metabolic impairments, which may explain why rising cases of childhood diabetes are associated with highly polluted areas.

## Conclusion and Future Perspectives

As the onset of metabolic disorders steadily increases in children and young adults, there is a great need to understand this etiology. Significant associations have been found between prenatal exposure to environmental pollutants and the heightened risk for metabolic impairments (69, 71, 73, 125). One potential mechanism is an increase in neuroinflammation, particularly affecting the hypothalamus. This is especially relevant considering the known neurotoxicity of air pollutants. Here, we propose a current gap highlighting the susceptibility of the hypothalamus during sensitive perinatal periods and how environmental insults may impact the hypothalamic programming of metabolism. Neuroinflammation may have a larger effect on hypothalamic development than previously thought, thus predisposing future generations to metabolic syndrome. Further research is needed to elucidate the molecular mechanisms that predispose offspring to metabolic disease. While it is clear that some particles and compounds can cross the placenta and have an impact on fetal development, the direct effect of these pollutants on hypothalamic development is unclear. Similarly, the direct or indirect impact of pollution-triggered maternal inflammation on the offspring’s metabolic health remains to be defined. While previous studies have assessed the outcomes of prenatal pollution on brain development, few have focused on the role of hypothalamic developmental circuits during fetal development on the later life metabolic outcomes. Finally, as research into prenatal pollution-induced neuroinflammation as a potential cause for metabolic dysfunction is limited, studies looking into therapeutic interventions remain scarce. Overall, significant challenges remain in understanding how pollution exposures impact fetal neurodevelopment and later life metabolism.

## Author Contributions

MS conceptualized the study, revised, and critically reviewed the manuscript. LK and SS drafted and revised the manuscript. GM critically reviewed the manuscript. All authors agree to be accountable for the content of the work. All authors contributed to the article and approved the submitted version.

## Funding

This study was supported by American Diabetes Association grant #1-lB-IDF-063, Michigan Diabetes Research Center P30-DK020572, CURES Center Grant (P30 ES020957), and NIEHS R01DK129681 for MS.

## Conflict of Interest

The authors declare that the research was conducted in the absence of any commercial or financial relationships that could be construed as a potential conflict of interest.

## Publisher’s Note

All claims expressed in this article are solely those of the authors and do not necessarily represent those of their affiliated organizations, or those of the publisher, the editors and the reviewers. Any product that may be evaluated in this article, or claim that may be made by its manufacturer, is not guaranteed or endorsed by the publisher.

## References

[1] Air Pollution. Available at: https://www.who.int/health-topics/air-pollution (Accessed 2022 Apr 27).

[2] Burden of Disease From the Joint Effects of Household and Ambient Air Pollution for 2016. Available at: https://www.ccacoalition.org/en/resources/burden-disease-joint-effects-household-and-ambient-air-pollution-2016 (Accessed 2022 Apr 27).

[3] Ambient (Outdoor) Air Pollution. Available at: https://www.who.int/news-room/fact-sheets/detail/ambient-(outdoor)-air-quality-and-health (Accessed 2022 Apr 26).

[4] Ambient Air Pollution Attributable Deaths. Available at: https://www.who.int/data/gho/data/indicators/indicator-details/GHO/ambient-air-pollution-attributable-deaths (Accessed 2022 Apr 27).

[5] US EPA O. Criteria Air Pollutants (2014). Available at: https://www.epa.gov/criteria-air-pollutants (Accessed 2022 Apr 27).

[6] Lemke LD Lamerato LE Xu X Booza JC Reiners JJ Raymond Iii DM. Geospatial Relationships of Air Pollution and Acute Asthma Events Across the Detroit-Windsor International Border: Study Design and Preliminary Results. J Expo Sci Env Epidemiol. (2014) 24(4):346–57. doi: 10.1038/jes.2013.78 PMC4063324 24220215

[7] Schraufnagel DE Balmes JR Cowl CT De Matteis S Jung SH Mortimer K. Air Pollution and Noncommunicable Diseases: A Review by the Forum of International Respiratory Societies’ Environmental Committee, Part 1: The Damaging Effects of Air Pollution. Chest (2019) 155(2):409–16. doi: 10.1016/j.chest.2018.10.042 PMC6904855 30419235

[8] Schraufnagel DE Balmes JR Cowl CT De Matteis S Jung SH Mortimer K. Air Pollution and Noncommunicable Diseases: A Review by the Forum of International Respiratory Societies’ Environmental Committee, Part 2: Air Pollution and Organ Systems. Chest (2019) 155(2):417–26. doi: 10.1016/j.chest.2018.10.041 PMC6904854 30419237

[9] Bala GP Rajnoveanu RM Tudorache E Motisan R Oancea C. Air Pollution Exposure-the (in)Visible Risk Factor for Respiratory Diseases. Env Sci pollut Res Int (2021) 28(16):19615–28. doi: 10.1007/s11356-021-13208-x PMC8099844 33660184

[10] Cipriani G Danti S Carlesi C Borin G. Danger in the Air: Air Pollution and Cognitive Dysfunction. Am J Alzheimers Demen. (2018) 33(6):333–41. doi: 10.1177/1533317518777859 PMC10852418 29874918

[11] Church JS Tijerina PB Emerson FJ Coburn MA Blum JL Zelikoff JT. Perinatal Exposure to Concentrated Ambient Particulates Results in Autism-Like Behavioral Deficits in Adult Mice. Neurotoxicology (2018) 65:231–40. doi: 10.1016/j.neuro.2017.10.007 PMC5857220 29104007

[12] Gorr MW Velten M Nelin TD Youtz DJ Sun Q Wold LE. Early Life Exposure to Air Pollution Induces Adult Cardiac Dysfunction. Am J Physiol Heart Circ Physiol (2014) 307(9):H1353–60. doi: 10.1152/ajpheart.00526.2014 PMC4217011 25172901

[13] Rosa MJ Hair GM Just AC Kloog I Svensson K Pizano-Zarate ML. Identifying Critical Windows of Prenatal Particulate Matter (PM2.5) Exposure and Early Childhood Blood Pressure. Env Res (2020) 182:109073. doi: 10.1016/j.envres.2019.109073 31881529 PMC7024649

[14] Fall CHD Kumaran K. Metabolic Programming in Early Life in Humans. Philos Trans R Soc B Biol Sci (2019) 374(1770):20180123. doi: 10.1098/rstb.2018.0123 PMC6460078 30966889

[15] Almeida DL Pavanello A Saavedra LP Pereira TS de Castro-Prado MAA de Freitas Mathias PC. Environmental Monitoring and the Developmental Origins of Health and Disease. J Dev Orig Health Dis (2019) 10(6):608–15. doi: 10.1017/S2040174419000151 31130151

[16] Lind L Lind PM Lejonklou MH Dunder L Bergman A Guerrero-Bosagna C. Uppsala Consensus Statement on Environmental Contaminants and the Global Obesity Epidemic. Env Health Perspect (2016) 124(5):A81–3. doi: 10.1289/ehp.1511115 PMC4858400 27135406

[17] Lind L Salihovic S Lampa E Lind PM. Mixture Effects of 30 Environmental Contaminants on Incident Metabolic Syndrome-A Prospective Study. Env Int (2017) 107:8–15. doi: 10.1016/j.envint.2017.06.005 28648904

[18] Ogurtsova K da Rocha Fernandes JD Huang Y Linnenkamp U Guariguata L Cho NH. IDF Diabetes Atlas: Global Estimates for the Prevalence of Diabetes for 2015 and 2040. Diabetes Res Clin Pr. (2017) 128:40–50. doi: 10.1016/j.diabres.2017.03.024 28437734

[19] Saklayen MG. The Global Epidemic of the Metabolic Syndrome. Curr Hypertens Rep (2018) 20(2):12. doi: 10.1007/s11906-018-0812-z 29480368 PMC5866840

[20] Global Report on Diabetes . Available at: https://www.who.int/publications-detail-redirect/9789241565257 (Accessed 2022 Apr 27).

[21] Koshiyama H Hamamoto Y Honjo S Wada Y Lkeda H. Hypothalamic Pathogenesis of Type 2 Diabetes. Med Hypotheses. (2006) 67(2):307–10. doi: 10.1016/j.mehy.2006.02.033 16616435

[22] Timper K Bruning JC. Hypothalamic Circuits Regulating Appetite and Energy Homeostasis: Pathways to Obesity. Model Mech (2017) 10(6):679–89. doi: 10.1242/dmm.026609 PMC5483000 28592656

[23] Myers MG Olson DP. Central Nervous System Control of Metabolism. Nature (2012) 491(7424):357–63. doi: 10.1038/nature11705 23151578

[24] Saper CB Lowell BB. The Hypothalamus. Curr Biol (2014) 24(23):R1111–6. doi: 10.1016/j.cub.2014.10.023 25465326

[25] Zeltser LM. Feeding Circuit Development and Early-Life Influences on Future Feeding Behaviour. Nat Rev Neurosci (2018) 19(5):302–16. doi: 10.1038/nrn.2018.23 PMC6347024 29662204

[26] MacKay H Abizaid A. Embryonic Development of the Hypothalamic Feeding Circuitry: Transcriptional, Nutritional, and Hormonal Influences. Mol Metab (2014) 3(9):813–22. doi: 10.1016/j.molmet.2014.09.004 PMC4264037 25506547

[27] McMillen IC Adam CL Mühlhäusler BS. Early Origins of Obesity: Programming the Appetite Regulatory System. J Physiol (2005) 565(1):9–17. doi: 10.1113/jphysiol.2004.081992 15705647 PMC1464497

[28] Shimada M Nakamura T. Time of Neuron Origin in Mouse Hypothalamic Nuclei. Exp Neurol (1973) 41(1):163–73. doi: 10.1016/0014-4886(73)90187-8 4743483

[29] Bouret SG. Nutritional Programming of Hypothalamic Development: Critical Periods and Windows of Opportunity. Int J Obes Suppl. (2012) 2(Suppl 2):S19–24. doi: 10.1038/ijosup.2012.17 PMC4850605 27152149

[30] Schwartz MW Seeley RJ Campfield LA Burn P Baskin DG. Identification of Targets of Leptin Action in Rat Hypothalamus. J Clin Invest. (1996) 98(5):1101–6. doi: 10.1172/JCI118891 PMC507530 8787671

[31] Myers MG. Leptin Receptor Signaling and the Regulation of Mammalian Physiology. Recent Prog Horm Res (2004) 59:287–304. doi: 10.1210/rp.59.1.287 14749507

[32] Guan HZ Dong J Jiang ZY Chen X. α-MSH Influences the Excitability of Feeding-Related Neurons in the Hypothalamus and Dorsal Vagal Complex of Rats. BioMed Res Int (2017) 2017:2034691. doi: 10.1155/2017/2034691 29318141 PMC5727559

[33] Michaud J. The Developmental Program of the Hypothalamus and its Disorders. Clin Genet (2001) 60(4):255–63. doi: 10.1034/j.1399-0004.2001.600402.x 11683768

[34] Rosin JM Vora SR Kurrasch DM. Depletion of Embryonic Microglia Using the CSF1R Inhibitor PLX5622 has Adverse Sex-Specific Effects on Mice, Including Accelerated Weight Gain, Hyperactivity and Anxiolytic-Like Behaviour. Brain Behav Immun (2018) 73:682–97. doi: 10.1016/j.bbi.2018.07.023 30056204

[35] Bolton JL Huff NC Smith SH Mason SN Foster WM Auten RL. Maternal Stress and Effects of Prenatal Air Pollution on Offspring Mental Health Outcomes in Mice. Environ Health Perspect (2013) 121(9):1075–82. doi: 10.1289/ehp.1306560 PMC3764088 23823752

[36] Bolton JL Marinero S Hassanzadeh T Natesan D Le D Belliveau C. Gestational Exposure to Air Pollution Alters Cortical Volume, Microglial Morphology, and Microglia-Neuron Interactions in a Sex-Specific Manner. Front Synaptic Neurosci (2017) 9:10. doi: 10.3389/fnsyn.2017.00010 28620294 PMC5449437

[37] Levesque S Taetzsch T Lull ME Kodavanti U Stadler K Wagner A. Diesel Exhaust Activates and Primes Microglia: Air Pollution, Neuroinflammation, and Regulation of Dopaminergic Neurotoxicity. Environ Health Perspect (2011) 119(8):1149–55. doi: 10.1289/ehp.1002986 PMC3237351 21561831

[38] Levesque S Taetzsch T Lull ME Johnson JA McGraw C Block ML. The Role of MAC1 in Diesel Exhaust Particle-Induced Microglial Activation and Loss of Dopaminergic Neuron Function. J Neurochem (2013) 125(5):756–65. doi: 10.1111/jnc.12231 PMC3660420 23470120

[39] Rosin JM Kurrasch DM. Bisphenol A and Microglia: Could Microglia be Responsive to This Environmental Contaminant During Neural Development? Am J Physiol Endocrinol Metab (2018) 315(2):E279–85. doi: 10.1152/ajpendo.00443.2017 29812986

[40] Gomez-Budia M Konttinen H Saveleva L Korhonen P Jalava PI Kanninen KM. Glial Smog: Interplay Between Air Pollution and Astrocyte-Microglia Interactions. Neurochem Int (2020) 136:104715. doi: 10.1016/j.neuint.2020.104715 32169588

[41] Hanamsagar R Bilbo SD. Environment Matters: Microglia Function and Dysfunction in a Changing World. Curr Opin Neurobiol (2017) 47:146–55. doi: 10.1016/j.conb.2017.10.007 PMC5732848 29096243

[42] Jayaraj RL Rodriguez EA Wang Y Block ML. Outdoor Ambient Air Pollution and Neurodegenerative Diseases: The Neuroinflammation Hypothesis. Curr Environ Health Rep (2017) 4(2):166–79. doi: 10.1007/s40572-017-0142-3 28444645

[43] Li Q Barres BA. Microglia and Macrophages in Brain Homeostasis and Disease. Nat Rev Immunol (2018) 18(4):225–42. doi: 10.1038/nri.2017.125 29151590

[44] Thion MS Ginhoux F Garel S. Microglia and Early Brain Development: An Intimate Journey. Science (2018) 362(6411):185–9. doi: 10.1126/science.aat0474 30309946

[45] Matcovitch-Natan O Winter DR Giladi A Vargas Aguilar S Spinrad A Sarrazin S. Microglia Development Follows a Stepwise Program to Regulate Brain Homeostasis. Science (2016) 353(6301):aad8670. doi: 10.1126/science.aad8670 27338705

[46] Elson AE Simerly RB. Developmental Specification of Metabolic Circuitry. Front Neuroendocrinol. (2015) 39:38–51. doi: 10.1016/j.yfrne.2015.09.003 26407637 PMC4681622

[47] Menassa DA Gomez-Nicola D. Microglial Dynamics During Human Brain Development. Front Immunol (2018) 9:1014. doi: 10.3389/fimmu.2018.01014 29881376 PMC5976733

[48] Schulz C Gomez Perdiguero E Chorro L Szabo-Rogers H Cagnard N Kierdorf K. A Lineage of Myeloid Cells Independent of Myb and Hematopoietic Stem Cells. Science (2012) 336(6077):86–90. doi: 10.1126/science.1219179 22442384

[49] Harry GJ Kraft AD. Microglia in the Developing Brain: A Potential Target With Lifetime Effects. NeuroToxicology (2012) 33(2):191–206. doi: 10.1016/j.neuro.2012.01.012 22322212 PMC3299893

[50] Bordeleau M Lacabanne C Fernandez de Cossio L Vernoux N Savage JC Gonzalez-Ibanez F. Microglial and Peripheral Immune Priming is Partially Sexually Dimorphic in Adolescent Mouse Offspring Exposed to Maternal High-Fat Diet. J Neuroinflammation. (2020) 17(1):264. doi: 10.1186/s12974-020-01914-1 32891154 PMC7487673

[51] Haley MJ Brough D Quintin J Allan SM. Microglial Priming as Trained Immunity in the Brain. Neuroscience (2019) 405:47–54. doi: 10.1016/j.neuroscience.2017.12.039 29292078

[52] Kracht L Borggrewe M Eskandar S Brouwer N Chuva de Sousa Lopes SM Laman JD. Human Fetal Microglia Acquire Homeostatic Immune-Sensing Properties Early in Development. Science (2020) 369(6503):530–7. doi: 10.1126/science.aba5906 32732419

[53] Reemst K Noctor SC Lucassen PJ Hol EM. The Indispensable Roles of Microglia and Astrocytes During Brain Development. Front Hum Neurosci (2016) 10:566. doi: 10.3389/fnhum.2016.00566 27877121 PMC5099170

[54] Marsters CM Nesan D Far R Klenin N Pittman QJ Kurrasch DM. Embryonic Microglia Influence Developing Hypothalamic Glial Populations. J Neuroinflammation. (2020) 17(1):146. doi: 10.1186/s12974-020-01811-7 32375817 PMC7201702

[55] Rosin JM Marsters CM Malik F Far R Adnani L Schuurmans C. Embryonic Microglia Interact With Hypothalamic Radial Glia During Development and Upregulate the TAM Receptors MERTK and AXL Following an Insult. Cell Rep (2021) 34(1):108587. doi: 10.1016/j.celrep.2020.108587 33406432

[56] Chaboub LS Deneen B. Developmental Origins of Astrocyte Heterogeneity: The Final Frontier of CNS Development. Dev Neurosci (2012) 34(5):379–88. doi: 10.1159/000343723 PMC3576470 23147551

[57] Antony JM Paquin A Nutt SL Kaplan DR Miller FD. Endogenous Microglia Regulate Development of Embryonic Cortical Precursor Cells. J Neurosci Res (2011) 89(3):286–98. doi: 10.1002/jnr.22533 21259316

[58] Matejuk A Ransohoff RM. Crosstalk Between Astrocytes and Microglia: An Overview. Front Immunol (2020) 11:1416. doi: 10.3389/fimmu.2020.01416 32765501 PMC7378357

[59] Stogsdill JA Ramirez J Liu D Kim YH Baldwin KT Enustun E. Astrocytic Neuroligins Control Astrocyte Morphogenesis and Synaptogenesis. Nature (2017) 551(7679):192–7. doi: 10.1038/nature24638 PMC5796651 29120426

[60] Jones EV Bernardinelli Y Tse YC Chierzi S Wong TP Murai KK. Astrocytes Control Glutamate Receptor Levels at Developing Synapses Through SPARC-Beta-Integrin Interactions. J Neurosci (2011) 31(11):4154–65. doi: 10.1523/JNEUROSCI.4757-10.2011 PMC6623508 21411656

[61] Ricci G Volpi L Pasquali L Petrozzi L Siciliano G. Astrocyte-Neuron Interactions in Neurological Disorders. J Biol Phys (2009) 35(4):317–36. doi: 10.1007/s10867-009-9157-9 PMC2750745 19669420

[62] Bixel MG Hamprecht B. Generation of Ketone Bodies From Leucine by Cultured Astroglial Cells. J Neurochem (1995) 65(6):2450–61. doi: 10.1046/j.1471-4159.1995.65062450.x 7595539

[63] Heithoff BP George KK Phares AN Zuidhoek IA Munoz-Ballester C Robel S. Astrocytes are Necessary for Blood-Brain Barrier Maintenance in the Adult Mouse Brain. Glia (2021) 69(2):436–72. doi: 10.1002/glia.23908 PMC7736206 32955153

[64] Spampinato SF Bortolotto V Canonico PL Sortino MA Grilli M. Astrocyte-Derived Paracrine Signals: Relevance for Neurogenic Niche Regulation and Blood-Brain Barrier Integrity. Front Pharmacol (2019) 10:1346. doi: 10.3389/fphar.2019.01346 31824311 PMC6881379

[65] Folick A Koliwad SK Valdearcos M. Microglial Lipid Biology in the Hypothalamic Regulation of Metabolic Homeostasis(2021) (Accessed 2022 May 31). 10.3389/fendo.2021.668396 PMC8191416 34122343

[66] Kim DW Washington PW Wang ZQ Lin SH Sun C Ismail BT. The Cellular and Molecular Landscape of Hypothalamic Patterning and Differentiation From Embryonic to Late Postnatal Development. Nat Commun (2020) 11:4360. doi: 10.1038/s41467-020-18231-z 32868762 PMC7459115

[67] Henn A Kirner S Leist M. TLR2 Hypersensitivity of Astrocytes as Functional Consequence of Previous Inflammatory Episodes. J Immunol (2011) 186(5):3237–47. doi: 10.4049/jimmunol.1002787 21282508

[68] Catale C Gironda S Lo Iacono L Carola V. Microglial Function in the Effects of Early-Life Stress on Brain and Behavioral Development. J Clin Med (2020) 9(2):468. doi: 10.3390/jcm9020468 PMC7074320 32046333

[69] Bolton JL Auten RL Bilbo SD. Prenatal Air Pollution Exposure Induces Sexually Dimorphic Fetal Programming of Metabolic and Neuroinflammatory Outcomes in Adult Offspring. Brain Behav Immun (2014) 37:30–44. doi: 10.1016/j.bbi.2013.10.029 24184474

[70] Purkayastha S Cai D. Neuroinflammatory Basis of Metabolic Syndrome. Mol Metab (2013) 2(4):356–63. doi: 10.1016/j.molmet.2013.09.005 PMC3854982 24327952

[71] Bolton JL Smith SH Huff NC Gilmour MI Foster WM Auten RL. Prenatal Air Pollution Exposure Induces Neuroinflammation and Predisposes Offspring to Weight Gain in Adulthood in a Sex-Specific Manner. FASEB J (2012) 26(11):4743–54. doi: 10.1096/fj.12-210989 22815382

[72] Hanamsagar R Bilbo SD. Sex Differences in Neurodevelopmental and Neurodegenerative Disorders: Focus on Microglial Function and Neuroinflammation During Development. J Steroid Biochem Mol Biol (2016) 160:127–33. doi: 10.1016/j.jsbmb.2015.09.039 PMC4829467 26435451

[73] Koshko L Debarba LK Sacla M de Lima JBM Didyuk O Fakhoury P. *In Utero* Maternal Benzene Exposure Predisposes to the Metabolic Imbalance in the Offspring. Toxicol Sci (2021) 180(2):252–61. doi: 10.1093/toxsci/kfab010 33502539

[74] Debarba LK Mulka A Lima JBM Didyuk O Fakhoury P Koshko L. Acarbose Protects From Central and Peripheral Metabolic Imbalance Induced by Benzene Exposure. Brain Behav Immun (2020) 89:87–99. doi: 10.1016/j.bbi.2020.05.073 32505715

[75] Tzschentke B Bogatyrev S Schellong K Rancourt RC Plagemann A. Temporary Prenatal Hyperglycemia Leads to Postnatal Neuronal “Glucose-Resistance” in the Chicken Hypothalamus. Brain Res (2015) 1618:231–40. doi: 10.1016/j.brainres.2015.05.037 26054304

[76] Steculorum SM Bouret SG. Maternal Diabetes Compromises the Organization of Hypothalamic Feeding Circuits and Impairs Leptin Sensitivity in Offspring. Endocrinology (2011) 152(11):4171–9. doi: 10.1210/en.2011-1279 PMC3199015 21862611

[77] Dickerson SM Cunningham SL Gore AC. Prenatal PCBs Disrupt Early Neuroendocrine Development of the Rat Hypothalamus. Toxicol Appl Pharmacol (2011) 252(1):36–46. doi: 10.1016/j.taap.2011.01.012 21277884 PMC3060304

[78] Bell MR Dryden A Will R Gore AC. Sex Differences in Effects of Gestational Polychlorinated Biphenyl Exposure on Hypothalamic Neuroimmune and Neuromodulator Systems in Neonatal Rats. Toxicol Appl Pharmacol (2018) 353:55–66. doi: 10.1016/j.taap.2018.06.002 29879404 PMC7846971

[79] MacKay H Patterson ZR Abizaid A. Perinatal Exposure to Low-Dose Bisphenol-A Disrupts the Structural and Functional Development of the Hypothalamic Feeding Circuitry. Endocrinology (2017) 158(4):768–77. doi: 10.1210/en.2016-1718 28323920

[80] Yam KY Ruigrok SR Ziko I De Luca SN Lucassen PJ Spencer SJ. Ghrelin and Hypothalamic NPY/AgRP Expression in Mice are Affected by Chronic Early-Life Stress Exposure in a Sex-Specific Manner. Psychoneuroendocrinology (2017) 86:73–7. doi: 10.1016/j.psyneuen.2017.09.006 28917185

[81] Nesan D Feighan KM Antle MC Kurrasch DM. Gestational Low-Dose BPA Exposure Impacts Suprachiasmatic Nucleus Neurogenesis and Circadian Activity With Transgenerational Effects. Sci Adv (2021) 7(22):eabd1159. doi: 10.1126/sciadv.abd1159 34049886 PMC8163075

[82] Rosin JM Tretiakov N Hanniman E Hampton K Kurrasch DM. Gestational Bisphenol A Exposure Impacts Embryonic Hypothalamic Microglia Numbers, Ramification, and Phagocytic Cups. Front Neurosci (2022) 16. doi: 10.3389/fnins.2022.830399 PMC8894877 35250464

[83] Takahashi M Komada M Miyazawa K Goto S Ikeda Y. Bisphenol A Exposure Induces Increased Microglia and Microglial Related Factors in the Murine Embryonic Dorsal Telencephalon and Hypothalamus. Toxicol Lett (2018) 284:113–9. doi: 10.1016/j.toxlet.2017.12.010 29248573

[84] Miranda RA Gaspar de Moura E Lisboa PC. Tobacco Smoking During Breastfeeding Increases the Risk of Developing Metabolic Syndrome in Adulthood: Lessons From Experimental Models. Food Chem Toxicol (2020) 144:111623. doi: 10.1016/j.fct.2020.111623 32738371

[85] Peixoto TC Moura EG Oliveira E Younes-Rapozo V Soares PN Rodrigues VST. Hypothalamic Neuropeptides Expression and Hypothalamic Inflammation in Adult Rats That Were Exposed to Tobacco Smoke During Breastfeeding: Sex-Related Differences. Neuroscience (2019) 418:69–81. doi: 10.1016/j.neuroscience.2019.08.006 31487543

[86] Younes-Rapozo V Moura EG Manhaes AC Pinheiro CR Santos-Silva AP de Oliveira E. Maternal Nicotine Exposure During Lactation Alters Hypothalamic Neuropeptides Expression in the Adult Rat Progeny. Food Chem Toxicol (2013) 58:158–68. doi: 10.1016/j.fct.2013.04.036 23623838

[87] de Oliveira E Moura EG Santos-Silva AP Pinheiro CR Lima NS Nogueira-Neto JF. Neonatal Nicotine Exposure Causes Insulin and Leptin Resistance and Inhibits Hypothalamic Leptin Signaling in Adult Rat Offspring. J Endocrinol (2010) 206(1):55–63. doi: 10.1677/JOE-10-0104 20453077

[88] Stojakovic A Espinosa EP Farhad OT Lutfy K. Effects of Nicotine on Homeostatic and Hedonic Components of Food Intake. J Endocrinol (2017) 235(1):R13–31. doi: 10.1530/JOE-17-0166 PMC5578410 28814527

[89] Younes-Rapozo V Moura EG Manhaes AC Pinheiro CR Carvalho JC Barradas PC. Neonatal Nicotine Exposure Leads to Hypothalamic Gliosis in Adult Overweight Rats. J Neuroendocr. (2015) 27(12):887–98. doi: 10.1111/jne.12328 26453898

[90] Rivest S Lacroix S Vallières L Nadeau S Zhang J Laflamme N. How the Blood Talks to the Brain Parenchyma and the Paraventricular Nucleus of the Hypothalamus During Systemic Inflammatory and Infectious Stimuli. Proc Soc Exp Biol Med Soc Exp Biol Med N Y N (2000) 223(1):22–38. doi: 10.1046/j.1525-1373.2000.22304.x 10632958

[91] Calderón-Garcidueñas L Mora-Tiscareño A Ontiveros E Gómez-Garza G Barragán-Mejía G Broadway J. Air Pollution, Cognitive Deficits and Brain Abnormalities: A Pilot Study With Children and Dogs. Brain Cogn. (2008) 68(2):117–27. doi: 10.1016/j.bandc.2008.04.008 18550243

[92] Mattei D Ivanov A Ferrai C Jordan P Guneykaya D Buonfiglioli A. Maternal Immune Activation Results in Complex Microglial Transcriptome Signature in the Adult Offspring That Is Reversed by Minocycline Treatment. Transl Psychiatry (2017) 7(5):e1120. doi: 10.1038/tp.2017.80 28485733 PMC5534948

[93] Han VX Patel S Jones HF Nielsen TC Mohammad SS Hofer MJ. Maternal Acute and Chronic Inflammation in Pregnancy Is Associated With Common Neurodevelopmental Disorders: A Systematic Review. Transl Psychiatry (2021) 11(1):1–12. doi: 10.1038/s41398-021-01198-w 33479207 PMC7820474

[94] Zawadzka A Cieślik M Adamczyk A. The Role of Maternal Immune Activation in the Pathogenesis of Autism: A Review of the Evidence, Proposed Mechanisms and Implications for Treatment. Int J Mol Sci (2021) 22(21):11516. doi: 10.3390/ijms222111516 34768946 PMC8584025

[95] Estes ML McAllister AK. Maternal Immune Activation: Implications for Neuropsychiatric Disorders. Science (2016) 353(6301):772–7. doi: 10.1126/science.aag3194 PMC5650490 27540164

[96] Baines KJ Hillier DM Haddad FL Rajakumar N Schmid S Renaud SJ. Maternal Immune Activation Alters Fetal Brain Development and Enhances Proliferation of Neural Precursor Cells in Rats(2020) (Accessed 2022 May 20). 10.3389/fimmu.2020.01145 PMC7295982 32582210

[97] Cassidy-Bushrow AE Burmeister C Birbeck J Chen Y Lamerato L Lemke LD. Ambient BTEX Exposure and Mid-Pregnancy Inflammatory Biomarkers in Pregnant African American Women. J Reprod Immunol (2021) 145:103305. doi: 10.1016/j.jri.2021.103305 33725526 PMC8164983

[98] Slama R Thiebaugeorges O Goua V Aussel L Sacco P Bohet A. Maternal Personal Exposure to Airborne Benzene and Intrauterine Growth. Env Health Perspect (2009) 117(8):1313–21. doi: 10.1289/ehp.0800465 PMC2721878 19672414

[99] Zahran S Weiler S Mielke HW Pena AA. Maternal Benzene Exposure and Low Birth Weight Risk in the United States: A Natural Experiment in Gasoline Reformulation. Env Res (2012) 112:139–46. doi: 10.1016/j.envres.2011.11.008 22177084

[100] Chen D Cho SI Chen C Wang X Damokosh AI Ryan L. Exposure to Benzene, Occupational Stress, and Reduced Birth Weight. Occup Env Med (2000) 57(10):661–7. doi: 10.1136/oem.57.10.661 PMC1739869 10984337

[101] Zambon A Rico LC Herman M Gundacker A Telalovic A Hartenberger LM. Gestational Immune Activation Disrupts Hypothalamic Neurocircuits of Maternal Care Behavior. Mol Psychiatry (2022). doi: 10.1038/s41380-022-01602-x PMC9112243 35581295

[102] Basil P Li Q Gui H Hui TCK Ling VHM Wong CCY. Prenatal Immune Activation Alters the Adult Neural Epigenome But can be Partly Stabilised by a N-3 Polyunsaturated Fatty Acid Diet. Transl Psychiatry (2018) 8:125. doi: 10.1038/s41398-018-0167-x 29967385 PMC6028639

[103] Wang S Yan JY Lo YK Carvey PM Ling Z. Dopaminergic and Serotoninergic Deficiencies in Young Adult Rats Prenatally Exposed to the Bacterial Lipopolysaccharide. Brain Res (2009) 1265:196–204. doi: 10.1016/j.brainres.2009.02.022 19236855

[104] Ingvorsen C Brix S Ozanne SE Hellgren LI. The Effect of Maternal Inflammation on Foetal Programming of Metabolic Disease. Acta Physiol (2015) 214(4):440–9. doi: 10.1111/apha.12533 26011013

[105] Burton GJ Fowden AL. The Placenta: A Multifaceted, Transient Organ. Philos Trans R Soc B Biol Sci (2015) 370(1663):20140066. doi: 10.1098/rstb.2014.0066 PMC4305167 25602070

[106] Ross EJ Graham DL Money KM Stanwood GD. Developmental Consequences of Fetal Exposure to Drugs: What We Know and What We Still Must Learn. Neuropsychopharmacology (2015) 40(1):61–87. doi: 10.1038/npp.2014.147 24938210 PMC4262892

[107] Dimasuay KG Boeuf P Powell TL Jansson T. Placental Responses to Changes in the Maternal Environment Determine Fetal Growth. Front Physiol (2016) 7:12. doi: 10.3389/fphys.2016.00012 26858656 PMC4731498

[108] Dutta A Khramtsova G Brito K Alexander D Mueller A Chinthala S. Household Air Pollution and Chronic Hypoxia in the Placenta of Pregnant Nigerian Women: A Randomized Controlled Ethanol Cookstove Intervention. Sci Total Environ (2018) 619–620:212–20. doi: 10.1016/j.scitotenv.2017.11.091 29149745

[109] Gomara B Herrero L Ramos JJ Mateo JR Fernandez MA Garcia JF. Distribution of Polybrominated Diphenyl Ethers in Human Umbilical Cord Serum, Paternal Serum, Maternal Serum, Placentas, and Breast Milk From Madrid Population, Spain. Env Sci Technol (2007) 41(20):6961–8. doi: 10.1021/es0714484 17993135

[110] Meijer L Weiss J Van Velzen M Brouwer A Bergman A Sauer PJ. Serum Concentrations of Neutral and Phenolic Organohalogens in Pregnant Women and Some of Their Infants in The Netherlands. Env Sci Technol (2008) 42(9):3428–33. doi: 10.1021/es702446p 18522129

[111] Myohanen K Vahakangas K. Foetal Exposure to Food and Environmental Carcinogens in Human Beings. Basic Clin Pharmacol Toxicol (2012) 110(2):101–12. doi: 10.1111/j.1742-7843.2011.00761.x 21740528

[112] Schönfelder G Wittfoht W Hopp H Talsness CE Paul M Chahoud I. Parent Bisphenol A Accumulation in the Human Maternal-Fetal-Placental Unit. Environ Health Perspect (2002) 110(11):A703–7. doi: 10.1289/ehp.021100703 PMC1241091 12417499

[113] Ikezuki Y Tsutsumi O Takai Y Kamei Y Taketani Y. Determination of Bisphenol A Concentrations in Human Biological Fluids Reveals Significant Early Prenatal Exposure. Hum Reprod (2002) 17(11):2839–41. doi: 10.1093/humrep/17.11.2839 12407035

[114] Gerona RR Woodruff TJ Dickenson CA Pan J Schwartz JM Sen S. Bisphenol-A (BPA), BPA Glucuronide, and BPA Sulfate in Mid-Gestation Umbilical Cord Serum in a Northern and Central California Population. Environ Sci Technol (2013) 47(21):10.1021/es402764d. doi: 10.1021/es402764d PMC3881559 23941471

[115] Dowty BJ Laseter JL Storer J. The Transplacental Migration and Accumulation in Blood of Volatile Organic Constituents. Pediatr Res (1976) 10(7):696–701. doi: 10.1203/00006450-197607000-00013 934736

[116] Block M. The Neuroinflammation Hypothesis Of Urban Air Pollution Effects In The Brain. Innov Aging. (2018) 2(Suppl 1):864. doi: 10.1093/geroni/igy023.3223

[117] Snow SJ Henriquez AR Costa DL Kodavanti UP. Neuroendocrine Regulation of Air Pollution Health Effects: Emerging Insights. Toxicol Sci Off J Soc Toxicol (2018) 164(1):9–20. doi: 10.1093/toxsci/kfy129 PMC6659011 29846720

[118] Guo B Guo Y Nima Q Feng Y Wang Z Lu R. Exposure to Air Pollution is Associated With an Increased Risk of Metabolic Dysfunction-Associated Fatty Liver Disease. J Hepatol (2022) 76(3):518–25. doi: 10.1016/j.jhep.2021.10.016 34883157

[119] Liu C Wang B Liu S Li S Zhang K Luo B. Type 2 Diabetes Attributable to PM2.5: A Global Burden Study From 1990 to 2019. Env Int (2021) 156:106725. doi: 10.1016/j.envint.2021.106725 34171589

[120] Balti EV Echouffo-Tcheugui JB Yako YY Kengne AP. Air Pollution and Risk of Type 2 Diabetes Mellitus: A Systematic Review and Meta-Analysis. Diabetes Res Clin Pr. (2014) 106(2):161–72. doi: 10.1016/j.diabres.2014.08.010 25262110

[121] Yang Y Guo Y Qian ZM Ruan Z Zheng Y Woodward A. Ambient Fine Particulate Pollution Associated With Diabetes Mellitus Among the Elderly Aged 50 Years and Older in China. Env pollut (2018) 243(Pt B):815–23. doi: 10.1016/j.envpol.2018.09.056 30243190

[122] Weinmayr G Hennig F Fuks K Nonnemacher M Jakobs H Mohlenkamp S. Long-Term Exposure to Fine Particulate Matter and Incidence of Type 2 Diabetes Mellitus in a Cohort Study: Effects of Total and Traffic-Specific Air Pollution. Env Health (2015) 14:53. doi: 10.1186/s12940-015-0031-x 26087770 PMC4479324

[123] Suryadhi MAH Suryadhi PAR Abudureyimu K Ruma IMW Calliope AS Wirawan DN. Exposure to Particulate Matter (PM2.5) and Prevalence of Diabetes Mellitus in Indonesia. Env Int (2020) 140:105603. doi: 10.1016/j.envint.2020.105603 32344253

[124] Najafi ML Zarei M Gohari A Haghighi L Heydari H Miri M. Preconception Air Pollution Exposure and Glucose Tolerance in Healthy Pregnant Women in a Middle-Income Country. Env Health (2020) 19(1):131. doi: 10.1186/s12940-020-00682-y 33298083 PMC7727159

[125] Miranda RA da Silva Franco CC Previate C Alves VS Francisco FA Moreira VM. Particulate Matter Exposure During Perinatal Life Results in Impaired Glucose Metabolism in Adult Male Rat Offspring. Cell Physiol Biochem (2018) 49(1):395–405. doi: 10.1159/000492901 30153661

